# Randomised prospective phase II trial in multiple brain metastases comparing outcomes between hippocampal avoidance whole brain radiotherapy with or without simultaneous integrated boost: HA-SIB-WBRT study protocol

**DOI:** 10.1186/s12885-020-07565-y

**Published:** 2020-10-30

**Authors:** Brendan Seng Hup Chia, Jing Yun Leong, Ashley Li Kuan Ong, Cindy Lim, Shi Hui Poon, Melvin Lee Kiang Chua, Kevin Lee Min Chua, Grace Kusumawidjaja, Eu Tiong Chua, Fuh Yong Wong, Tih Shih Lee

**Affiliations:** 1 Division of Radiation Oncology, National Cancer Centre Singapore, 11 Hospital Crescent, Singapore, 169610 Singapore; 2 Department of Adult Neuro-developmental Service, Institute of Mental Health Singapore, 10 Buangkok View, Singapore, 539747 Singapore; 3 Division of Clinical Trials and Epidemiological Sciences, National Cancer Centre Singapore, 11 Hospital Crescent, Singapore, 169610 Singapore; 4 Department of Psychiatry, Singapore General Hospital, Outram Road, Singapore, 169608 Singapore

**Keywords:** Whole brain radiotherapy, Hippocampal-avoidance whole brain radiotherapy, Brain metastases, Study protocol

## Abstract

**Background:**

Recent evidence supports hippocampal avoidance with whole brain radiotherapy (HA-WBRT) as the recommended treatment option in patients with good prognosis and multiple brain metastases as this results in better neurocognitive preservation compared to whole brain radiotherapy. However, there is often poor tumour control with this technique due to the low doses given. Stereotactic Radiosurgery (SRS), a form of focused radiotherapy which is given to patients who have a limited number of brain metastases, delivers a higher radiation dose to the metastases resulting in better target lesion control. With improvements in radiation technology, advanced dose-painting techniques now allow a simultaneous integrated boost (SIB) dose to lesions whilst minimising doses to the hippocampus to potentially improve brain tumour control and preserve cognitive outcomes. This technique is abbreviated to HA-SIB-WBRT or HA-WBRT+SIB.

**Methods:**

We hypothesise that the SIB in HA-SIB-WBRT (experimental arm) will result in better tumour control compared to HA-WBRT (control arm). This may also lead to better intracranial disease control as well as functional and survival outcomes. We aim to conduct a prospective randomised phase II trial in patients who have good performance status, multiple brain metastases (4–25 lesions) and a reasonable life expectancy (> 6 months). These patients will be stratified according to the number of brain metastases and randomised between the 2 arms. We aim for a recruitment of 100 patients from a single centre over a period of 2 years. Our primary endpoint is target lesion control. These patients will be followed up over the following year and data on imaging, toxicity, quality of life, activities of daily living and cognitive measurements will be collected at set time points. The results will then be compared across the 2 arms and analysed.

**Discussion:**

Patients with brain metastases are living longer. Maintaining functional independence and intracranial disease control is thus increasingly important. Improving radiotherapy treatment techniques could provide better control and survival outcomes whilst maintaining quality of life, cognition and functional capacity. This trial will assess the benefits and possible toxicities of giving a SIB to HA-WBRT.

**Trial registration:**

Clinicaltrials.gov identifier: NCT04452084. Date of registration 30th June 2020.

## Introduction

### Background

The management of brain metastases presents a significant challenge in oncology. Whole brain radiation therapy (WBRT) is widely given to improve neurological symptoms, stop brain metastasis progression and possibly prolong survival. Unfortunately, neurocognitive functional decline is reported in a significant number of patients who undergo WBRT at rates of 31–57% at 3 months and 48–89% at 1 year [1].

The hippocampus plays a key role in learning, memory and cognitive function [2–4]. The significance of hippocampi radiation in clinical studies is emerging. Gondi et al. showed that a dose of > 7.3Gy to at least 40% of the hippocampus resulted in a significant decline in delayed recall in adult brain cancer patients [5].

A recent phase 3 trial, NRG-CC001, randomised 518 patients to Hippocampal avoidance-WBRT (HA-WBRT) against standard WBRT. Cognitive endpoints were measured using a test battery of Hopkins Verbal Learning Test-Revised (HVLT-R), Controlled Oral Word Association, and Trail Making Test (TMT) Part A & B. At a median follow-up for alive patients of 7.9 months, a significant reduction in cognitive failure risk was noted (HR, 0.76; *P* = 0.03) in the HA-WBRT arm. This difference was most significant in the 4-month TMT Part B scores and the 6-month HVLT-R score. The median overall survival (OS) and progression free survival (PFS) did not differ and there was no increase in hippocampal relapses. However notably, OS and PFS in the HA-WBRT arm were poor at 6.3 months and 5.0 months respectively [6].

This trial introduced HA-WBRT as a new standard option in patients with multiple brain metastases and good prognosis. However, some clinicians would still favour the use of Stereotactic Radiosurgery (SRS) over HA-WBRT in patients with low volume brain metastases. The reasons could possibly be better cognitive sparing or tumoral control due to the higher ablative doses used in SRS. Several trials are underway comparing the use of HA-WBRT against SRS (ClinicalTrials.gov identifier: NCT03550391, NCT03075072 and NCT04277403).

### Rationale

In the landmark RTOG 95–08 trial which compared an SRS boost vs. no boost in patients undergoing WBRT, the patients in the boost arm had improved local control rates, better functional autonomy and reduced steroid need with few toxicities [7]. OS was also improved in some patients [8, 9]. With current dose-painting radiotherapy planning techniques, we are now able to deliver a simultaneous integrated boost (SIB) dose to the lesions while giving HA-WBRT, a technique abbreviated as HA-SIB-WBRT or HA-WBRT+SIB.

Several single-arm prospective studies looked at the use of WBRT+SIB or HA-SIB-WBRT in variable patient cohorts with SIB doses of 40–52.5Gy in 10–15 fractions [10–15]. The reported intracranial PFS was noted to be as high as 13.5 months with few ≥Grade 3 toxicities (≤6.5%), making it a potentially viable option. In a propensity score-matched comparison against WBRT, Popp et al. reported significantly improved local tumour control rates, intracranial PFS, reduction in neurological deaths and even better OS [12]. This has led the group to continue to a phase II HIPPORAD Trial comparing HA-WBRT+SIB against WBRT+SIB (German Clinical Trials Registry: DRKS00004598) [16].

Given that brain metastases are being detected earlier and better systemic options have extended the survival in many patients, the need to maintain functional independence and brain disease control is increasingly important. In the management of brain metastases, there is still an unmet need for patients with good prognoses but are unable to receive SRS to have better intracranial disease control over the new standard of HA-WBRT. It is thus important to assess if the addition of a SIB will improve the outcomes of HA-WBRT.

To our knowledge, there is no prospective trial underway comparing HA-SIB-WBRT against HA-WBRT. We thus aim to test if there is a clinical benefit for the additional SIB in HA-WBRT. In preparation for this trial, we performed a planning study to test the feasibility of dosimetric targets set for HA-SIB-WBRT in 5 patients with different tumour numbers, volumes and locations as well as experimented with various treatment planning techniques [17]. From this, we were then confident in our ability to proceed with our study.

We hypothesise that HA-SIB-WBRT will be able to increase both target tumour and intracranial disease control compared with HA-WBRT alone. This, therefore, has the potential to impact on survival outcomes whilst maintaining cognitive function and quality of life.

### Objectives

The purpose of the study is to assess the impact that adding SIB to standard of care treatment (HA-WBRT) has on local control, survival outcomes, cognition and other patient reported outcome measures (PROMs).

### Study design

This study will be conducted as a single centre prospective randomised phase II trial in patients with multiple brain metastases and good prognosis (> 6 months). The patients will be compared between the 2 arms: HA-WBRT (control) vs. HA-SIB-WBRT (experimental). The target recruitment is 100 patients over 2 years.

## Methods: participants, interventions and outcomes

### Study setting

**Fig. 1:**
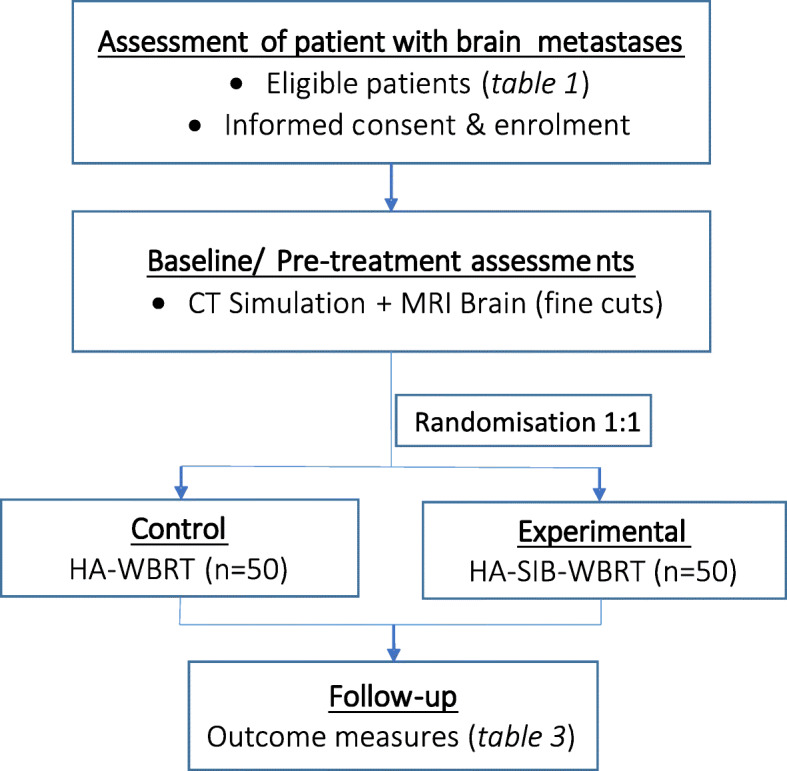
Project Schema

The trial will be conducted at the National Cancer Centre Singapore, which is the largest cancer centre in the country. The study workflow is depicted in Fig. 1.

### Eligibility criteria

Participants will be recruited if they meet the criteria set out in Table 1 below.

**Table 1:** Eligibility Criteria

Inclusion Criteria	Exclusion Criteria
• 21–80 years old patients • Radiological confirmed brain metastases (4–25 lesions) • Histologically proven malignancy • ECOG performance status ≤2 • Maximum lesion or cavity size ≤5 cm o For patients with large (≥3 cm) lesions, a neurosurgical consult is recommended due to the increased risk of cerebral oedema o If brain surgery or other invasive procedures are performed, the treatment should begin at least 2-weeks post-procedure • Life expectancy of at least 6 months • Negative serum pregnancy test within 14 days prior to registration for women of childbearing potential • Women of childbearing potential and male participants who are sexually active must agree to use a medically effective means of birth control throughout protocol treatment • Not suitable for or does not want SRS • Agrees to be randomised to either HA-WBRT or HA-SIB-WBRT	• Prior whole brain radiotherapy. o Prior SRS is not an exclusion. Details of treatment must be recorded. • Diffuse leptomeningeal disease • Extensive extracranial disease, not controlled by systemic treatment • Major medical or psychiatric illness, which in the investigator’s opinion would interfere with the completion of therapy and follow up • Dementia, ongoing psychotic episodes or moderate-severe depression (PHQ-9). • Recent stroke in the past 3 months • Symptomatic brain metastases limiting ADLs • Rapid progression of brain lesion • Patients unable to give informed consent • Total tumour planning target volume (PTV) > 60 cc • Radiological evidence of hydrocephalus • Contraindication to Gadolinium contrast-enhanced MRI brain • Patients with diagnoses of small cell carcinoma, lymphoma or primary brain tumour

### Interventions

The control arm in this study is HA-WBRT. This will be given 30Gy in 10 fractions.

The experimental procedure is HA-SIB-WBRT. 40-45Gy in 10 fractions will be used for the SIB boost depending on the location of the lesion. 45Gy was selected as it has a similar biological effective dose (BED 65.2Gy; α/β = 10) to the 21Gy SRS dose. 40Gy (EQD2 60Gy; α/β = 2) was selected for tumours within/ close to organs at risk (OARs) to meet the recommended dose limits of common OARs. Any post-operative cavity will be contoured using consensus guidelines [18]. The cavity will be given a boost of 40Gy due to the high risk of microscopic residual disease and tumour seeding. Any gross residual tumour should be boosted to 45Gy. Further radiotherapy dose guidance is described in Table 2.

**Table 2:** Radiotherapy Planning targets, Organs at Risk and Dose Guidance

Volume	Description	Dose Target/ Limits	Variation Acceptable	Notes
GTV_45Gy^b^	Contoured using fused contrast enhanced MRI images.	45Gy 98% covered by 100% dose Hotspot > 110% within GTV	95% covered by 95% dose	Lesion visible on at least 2 scan slices. Coverage to be comprised to meet OAR constraints
PTV_45Gy^b^	GTV + 0-2 mm isotropic margins	45Gy 98% covered by 95% dose	90% covered by 90% dose	Coverage to be comprised to meet OAR constraints
GTV_40Gy^b^	Tumour volume within/ ≤5 mm from OAR	40Gy 95% covered by 95% dose Hotspot > 110% within GTV	90% covered by 95% dose	Lesion visible on at least 2 scan slices. Coverage to be comprised to meet OAR constraints
PTV_40Gy^b^	PTV_40Gy = GTV_40Gy; i.e. no expansion	40Gy 95% covered by 95% dose	90% covered by 90% dose	Coverage to be comprised to meet OAR constraints
GTVall_5mm^b^	All GTVs + 5 mm isotropic margins	–	–	For plan optimisation
Left & Right Hippocampus	Contoured using fused MRI as per RTOG contouring atlas (http://www.rtog.org//corelab/contouringatlases/hippocampalsparing.aspx), excluding any GTV	D100% (Dose to 100% volume) ≤ 9Gy Dmax ≤16Gy Dmax ≤33Gy^a^	D100% ≤ 10Gy Dmax ≤17Gy Dmax ≤44Gy^ab^	Dmax to 0.03 cc
Hippocampal Avoidance Zone (HAZ)	Hippocampus + 5 mm isotropic margins	–	–	For plan optimization
Left & Right Optic chiasm	–	Dmax ≤33Gy	Dmax ≤37.5Gy^b^ Dmax ≤40Gy^ab^	Dmax to 0.03 cc
Left & Right Optic nerve	–	Dmax ≤33Gy	Dmax ≤35Gy^b^	Dmax to 0.03 cc
Left & Right Orbits	–	Dmax ≤33Gy	Dmax ≤35Gy^b^	Dmax to 0.03 cc
Left & Right Lens	–	Dmax ≤6Gy	Dmax ≤10Gy^b^	Dmax to 0.03 cc
Brain Stem	–	Dmax ≤33Gy	Dmax ≤37.5Gy^b^ Dmax ≤40Gy^ab^	Dmax to 0.03 cc
Left & Right Cochlear	–	Dmax ≤33Gy	Dmax ≤35Gy^a^	Dmax to 0.03 cc
Brain	Brain parenchyma down to cranial margin of dens	30Gy	–	–
PTV_Brain	(Brain + 3 mm isotropic margins) – (GTVall_5mm^b^ + HAZ)	95% of volume covered by 30Gy D2% ≤ 37.5Gy D98% (Dose to 98% of volume) ≥ 25Gy	90% of volume covered by 30Gy D98% ≥ 22.5Gy^b^ D2% ≤ 40Gy^b^	–

^a^*Applicable if tumours within/ ≤5 mm from OARs*^b^*Only applicable to HA-SIB-WBRT plans*

Patients whose plans are unable to meet the recommended constraints will not be eligible for the trial and will be dropped out and undergo WBRT or HA-WBRT as standard of care treatment. Patients can also voluntarily or involuntarily drop-out of the trial at any time after enrolment. These patients will not be replaced. The reason for drop-out must be recorded.

All treatment plans in the experimental arm will be reviewed and approved at the weekly Neuro-radiation oncology team audits prior to the start of treatment. Immobilisation will be performed in all patients. Daily image verification by image-guided radiotherapy is required.

All concomitant treatments should be documented. The use of concurrent cytotoxic systemic treatments is not allowed as this could cause additional or unexpected neuro-toxicities. If the participant is on systemic treatments, a treatment break of at least 7 days for immunotherapy or chemotherapy and 3 days for targeted therapy is recommended before and after radiotherapy. Interruptions should be discussed with the patient’s prescribing medical oncologist.

The use of dexamethasone during radiotherapy is not mandatory but is recommended for use if the patient with symptomatic brain metastases, significant cerebral oedema, large tumours or posterior cranial fossa lesions. The concurrent use of memantine for cognitive protection is recommended but not mandated.

### Outcomes and timeline

#### Primary endpoint

- Target lesion control
  ▪ Response to treated lesions will be rated based on RANO-Criteria [19] or RECIST 1.1-Criteria.

#### Secondary endpoints

- Intracranial Progression
  ▪ Target lesion or distal brain lesion
  ▪ Symptomatic or asymptomatic brain lesion
  - PFS
  - OS
    ▪ Neurological or non-neurological death
    ▪ Cancer or non-cancer related death
  - Cognitive Function
    ▪ HVLT-R (immediate recall, delayed recall, and total recall)
    ▪ Colour Trail Test (CTT) – a language-free version of the TMT
  - Quality of Life (QoL)
    ▪ Functional Assessment of Cancer Therapy with Brain Subscale (FACT-BR)
    ▪ Euro QOL – 5 Dimension – 5 Level (EQ-5D-5L)
  - Activities of Daily Living (ADL)
    ▪ Barthel Index of ADLs
  - Toxicity
    ▪ Scored using Common Terminology Criteria for Adverse Events (CTCAE) ver5.0 criteria
    ▪ Presence of radiation necrosis (asymptomatic or symptomatic)

All time points will be taken from the time of randomisation to event. If response assessment of the target lesion(s) is uncertain or equivocal, this should be reassessed by a multi-disciplinary team for clarification. When needed, this may be followed up with advanced imaging or histology.

Cognitive function, ADL and QoL will be assessed using patient-directed questionnaires that have been validated, available in local languages and used in the trials mentioned earlier for ease of comparison. These tests are optional but encouraged for all patients and will be conducted by a trained physician or clinical research associate at specified time points (Table 3). The total duration of the combined test is estimated to be 30 min. In the analysis of cognitive function, ADL and QoL, each patient will serve as his/her own control. The test results at each follow-up (post-treatment) time point will be compared to the baseline (pre-treatment) test result.

**Table 3:**
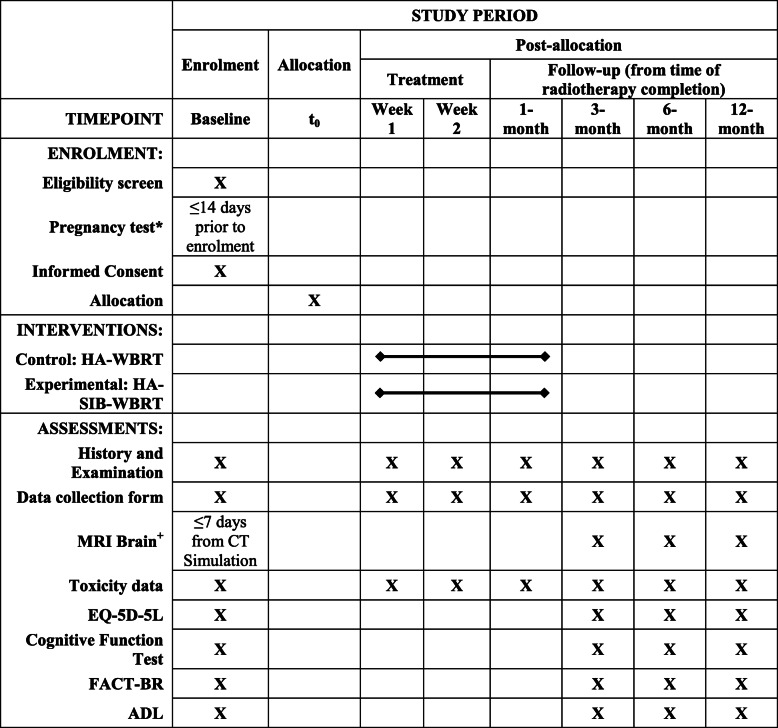
Schedule of Enrolment, Interventions, and Assessments Recommended Follow-up Schedule Protocol^

^The follow-up schedule is based on current recommended routine practice. A window period of +/− 2 weeks is allowed for the 1st month follow up visit. +/− 1 month is allowed for the 3rd, 6th and 12th month follow-up visit. It is advised that this be followed for standardisation of records, however individual deviations will be allowed. ^+^MRI Brain imaging if performed following surgery should be done 2-weeks post op. MRI is recommended for follow-up brain imaging but contrasted-CT scan is allowed

*Only in females with child-bearing potential

### Sample size

Assuming a 6-month target lesion control rate of 50% in the HA-WBRT arm, we will need 60 subjects (30 per arm) to detect a hazard ratio of 0.27 [9] between the 2 arms with 80% power using a 2-sided log-rank test at 5% significance level. A hazard ratio of 0.27 means the 6-month target lesion control rate in the HA-SIB-WBRT arm is expected to be 83%. These calculations assume that subjects will be followed up till target lesion progression, or for a minimum of 6 months. Assuming a 40% non-evaluable rate, an estimated total sample size of 100 subjects will be required.

### Assignment of interventions

Patients recruited will be stratified by the number of brain metastases < 10 vs. ≥10. Within each stratum, patients will be randomized in a 1:1 ratio to control vs. experimental arms. Simple randomization will be performed using a computer-generated random sequence allocation. This will be performed by the research coordinator after informed consent has been taken. The allocation sequence is concealed from the investigators. Patients and treating radiation oncologist will not be blinded to the intervention as it was opined that our patients would want to be aware of the treatment received and that unblinding would not significantly affect our primary objective measure.

## Data collection and management

### Data management

Data (soft-copy) will be kept in a password protected database “SingHealth REDCap”. All documented (hard copy) data including consent forms, data collection forms and neurocognitive, functional and ADL assessment forms will be stored in a secured research folder. Research coordinators will help ensure per-protocol follow-up and filling-in of data targets. 3-monthly random checks on the data will be performed by the investigators to ensure integrity and quality.

## Statistical methods

### Statistical analysis plans

Efficacy analyses will be performed using the intention-to-treat principle. Per-protocol analyses may be performed as secondary analyses. Safety analyses will include all patients who started treatment and will be performed according to the actual treatment received.

The primary endpoint, time to target lesion progression, will be defined as the time from randomisation to target lesion progression. Patients who pass away before documented target lesion progression will be censored at the last brain imaging assessment. Time to target lesion progression will be compared between the 2 treatment groups using a 2-sided log-rank test.

Time to intracranial progression, time to symptomatic brain progression, progression-free survival and overall survival will be defined as the time from randomisation to intracranial progression, symptomatic brain progression, overall progression and death from any cause respectively. All time-to-event endpoints will be summarized using the Kaplan-Meier method. Treatment arms will be compared using log-rank tests and Cox proportional hazards models, adjusting for the stratification variable (number of brain metastases). Other prognostic variables such as histology, ECOG status and disease-specific GPA class may also be included in the Cox models.

Adverse events will be recorded according to CTCAE version 5.0 and summarised by treatment arm.

Cognitive test (HVLT-R and CTT) scores will be standardised based on published norms: (Patient value – Published-norm mean value) ÷ Published-norm standard deviation value. Cognitive deterioration will be defined as a decline of at least 1 SD in score from baseline. FACT-BR scores will be transformed to a 0- to 100- point scale. A 10-point decrease will be considered clinically significant. Deterioration in functional independence will be defined as a decline of at least 10% in the Barthel ADL index from baseline.

The proportions of patients with cognitive deterioration, QoL deterioration and functional independence deterioration will be compared between treatment arms using Fisher’s exact test. A sensitivity analysis will be conducted assuming patients who had not completed the neurocognitive assessment or had passed away prior to the assessment time-point, had cognitive deterioration. Change from baseline values will be compared using 2-sample t-tests and repeated measures linear regression, adjusting for the stratification variable (number of brain metastases).

All analyses will use a 2-sided 0.05 level of significance. There will be no adjustment for multiple comparisons.

There is no planned interim analysis for this study.

## Oversight and monitoring

### Safety

There is no independent Data Safety Monitoring Committee for this study. Any ≥Grade 3 serious adverse events must be reviewed by the co-investigators to determine if this toxicity is treatment related. All serious treatment related toxicities must be reported to the institutional review board (IRB). If any ≥Grade 4 toxicities are noted, the study will stop for review of safety before continuation, cessation or amendment of trial.

The principal investigator and co-investigators will be responsible for monitoring patient recruitment, toxicities, observed results and the evaluation of data quality. This will be done every 3 months.

## Ethics and dissemination

### Research ethics

This study will be conducted in accordance with the ethical principles that have their origin in the Declaration of Helsinki and that are consistent with the Good Clinical Practice and the applicable regulatory requirements.

The Clinical Trial Protocol, including the Participant Information Sheet and Consent Form, has been approved by the SingHealth Centralised Institutional Review Board No. 2019/2407 prior to enrolment of any patient into the study.

Only the study team radiation oncology investigators will be allowed to take inform consent from potential trial participants.

### Confidentiality

Only the Principal investigator and study coordinators will have access to the research data. At the completion of the study, participant identifiers will be removed from stored data and anonymized. Re-identification of participants will be kept by a 3rd party person not involved in the study project. The participants’ name will not be used in any public report of the study.

## Discussion

The optimal management of patients with multiple brain metastases is multifaceted and depends on several factors including tumour type, volume and number of brain metastases, available brain penetrating drugs, prognosis and performance status. Traditionally, the treatment was limited to WBRT. Fortunately, improvements in technology and pharmacology have expanded the therapeutic options for these patients.

SRS which is often given in preference over WBRT in limited brain metastases (≤3–4 lesions) has shown better learning and memory preservation in several randomised trials [20]. In patients with > 4 brain lesions, WBRT remains the standard of care although the role of SRS is fast emerging. A prospective observational study and a large retrospective multi-institutional study have shown that the total number of brain metastases (up to 15 lesions) treated with SRS did not seem to affect the survival outcome and could be considered instead of WBRT [21, 22]. Despite this, there remains some controversy in giving SRS for multiple brain metastases. One would expect that with more brain metastases, the risk of brain micrometastases and intracranial failure is higher and thus giving SRS instead of WBRT would mean the need for early salvage treatment thus negating the clinical benefit [20, 23]. The previous SRS trials also did not perform comparisons against newer neurocognitive protecting strategies like HA-WBRT and the use of neuroprotective agents like memantine. Several randomised phase III trials are thus underway that will hopefully provide further clarity on the role of SRS in the setting of multiple brain metastases.

The NRG-CC001 trial which proved the benefit of HA-WBRT over WBRT had a poor intracranial PFS of 5.0 months [6]. This was not surprising, given the inherent low doses utilised in standard HA-WBRT. Several studies have shown that higher doses to target lesions could improve on local control rates and potentially reduce intracranial failure [9, 20, 23]. In turn, improving intracranial control could possibly result in improved performance status and reduced neurological death [8, 9, 12]. It would thus seem reasonable to consider giving tumours a simultaneous higher dose during HA-WBRT using HA-SIB-WBRT. The preliminary evidence for this has been promising. However, there is still a lack of high-level evidence on the benefit and safety it has over standard HA-WBRT. This trial thus sets out to explore the magnitude of these benefits including control rates, toxicities, survival outcomes, cognition and other PROMs. To the best of our knowledge, this is the only prospective, randomised trial comparing HA-WBRT against HA-SIB-WBRT. We, therefore, believe that this trial is significant as it will provide the evidence required to support its use.

## Trial status

The first patient was recruited on June 2020. The trial is currently ongoing and recruiting patients. The trial is registered on ClinicalTrials.gov under NCT04452084.

## Data Availability

Not applicable.

## Abbreviations

ADL: Activities of daily living
BED: Radiation biological effective dose
CT: Computer tomography
CTCAE: Common terminology criteria for adverse events
CTT: Colour trail test
ECOG: Eastern cooperative oncology group performance status
EQD2: Radiation equivalent dose in 2gy fractions
EQ-5D-5L: Euro QOL – 5 dimension – 5 level questionnaire
FACT-BR: Functional assessment of cancer therapy with brain subscale
GPA: Graded prognostic assessment prognostic index
GTV: Gross tumour volume
Gy: Gray
HAZ: Hippocampal avoidance zone.
HA-WBRT: Hippocampal avoidance whole brain radiotherapy
HA-SIB-WBRT: Hippocampal avoidance with simultaneous integrated boost whole brain radiotherapy
HVLT-R: Hopkins verbal learning test-revised
IRB: Institutional review board
MRI: Magnetic resonance imaging
OAR: Organs at risk
OS: Overall survival
PFS: Progression free survival
PROMs: Patient report outcome measures
PTV: Planning target volume
QoL: Quality of life
RANO: Response assessment in neuro-oncology
RECIST 1.1: Response evaluation criteria in solid tumours version 1.1
SIB: Simultaneous integrated boost
SRS: Stereotactic radiosurgery
TMT: Trail making test
WBRT: Whole brain radiotherapy
WBRT+SIB: Whole brain radiotherapy with simultaneous integrated boost
